# Genetic architecture and major genes for backfat thickness in pig lines of diverse genetic backgrounds

**DOI:** 10.1186/s12711-021-00671-w

**Published:** 2021-09-22

**Authors:** Miguel Gozalo-Marcilla, Jaap Buntjer, Martin Johnsson, Lorena Batista, Federico Diez, Christian R. Werner, Ching-Yi Chen, Gregor Gorjanc, Richard J. Mellanby, John M. Hickey, Roger Ros-Freixedes

**Affiliations:** 1 The Roslin Institute, The University of Edinburgh, Midlothian, UK; 2 The Royal (Dick) School of Veterinary Studies, The University of Edinburgh, Midlothian, UK; 3 Department of Animal Breeding and Genetics, Swedish University of Agricultural Sciences, Uppsala, Sweden; 4 The Pig Improvement Company, Genus plc, Hendersonville, TN USA; 5 Departament de Ciència Animal, Universitat de Lleida - Agrotecnio-CERCA Center, Lleida, Spain

## Abstract

**Background:**

Backfat thickness is an important carcass composition trait for pork production and is commonly included in swine breeding programmes. In this paper, we report the results of a large genome-wide association study for backfat thickness using data from eight lines of diverse genetic backgrounds.

**Methods:**

Data comprised 275,590 pigs from eight lines with diverse genetic backgrounds (breeds included Large White, Landrace, Pietrain, Hampshire, Duroc, and synthetic lines) genotyped and imputed for 71,324 single-nucleotide polymorphisms (SNPs). For each line, we estimated SNP associations using a univariate linear mixed model that accounted for genomic relationships. SNPs with significant associations were identified using a threshold of p < 10^–6^ and used to define genomic regions of interest. The proportion of genetic variance explained by a genomic region was estimated using a ridge regression model.

**Results:**

We found significant associations with backfat thickness for 264 SNPs across 27 genomic regions. Six genomic regions were detected in three or more lines. The average estimate of the SNP-based heritability was 0.48, with estimates by line ranging from 0.30 to 0.58. The genomic regions jointly explained from 3.2 to 19.5% of the additive genetic variance of backfat thickness within a line. Individual genomic regions explained up to 8.0% of the additive genetic variance of backfat thickness within a line. Some of these 27 genomic regions also explained up to 1.6% of the additive genetic variance in lines for which the genomic region was not statistically significant. We identified 64 candidate genes with annotated functions that can be related to fat metabolism, including well-studied genes such as *MC4R*, *IGF2*, and *LEPR*, and more novel candidate genes such as *DHCR7*, *FGF23*, *MEDAG*, *DGKI*, and *PTN*.

**Conclusions:**

Our results confirm the polygenic architecture of backfat thickness and the role of genes involved in energy homeostasis, adipogenesis, fatty acid metabolism, and insulin signalling pathways for fat deposition in pigs. The results also suggest that several less well-understood metabolic pathways contribute to backfat development, such as those of phosphate, calcium, and vitamin D homeostasis.

**Supplementary Information:**

The online version contains supplementary material available at 10.1186/s12711-021-00671-w.

## Background

Pork accounts for 35% of meat consumption worldwide, representing an important component of many human diets [1]. To align production with consumer demands, one of the key objectives in pig breeding programmes is the reduction of carcass fatness, resulting in increased growth efficiency and lean meat content [2]. This is typically achieved by including backfat thickness in the economic index for selection within pig lines. Backfat thickness is a good indirect predictor of overall body fat content, can be measured on the live animal by ultrasound, and has a high heritability [3–6].

Over the last two decades, there has been great interest in identifying candidate genes that regulate backfat thickness. Prominent genes that were discovered by linkage analysis include *IGF2* [7–9], *MC4R* [10, 11], and *LEPR* [12]. Since then, more than 1400 quantitative trait loci (QTL) related to backfat thickness have been reported (https://www.animalgenome.org/QTLdb). Results from these studies showed that backfat thickness is a polygenic trait that is regulated by a large number of small-effect variants. With the advent of single-nucleotide polymorphism (SNP) genotyping arrays, gene expression analyses, and other high-throughput genotyping technologies, many more candidate genes for backfat thickness have been reported that are involved in very diverse biological functions and metabolic pathways, such as: adipogenesis [13, 14]; lipid metabolism (biosynthesis, absorption, transport, catabolism and homeostasis) pathways, including those related to fatty acids and triglycerides [13, 15, 16]; regulation of feed intake and energy homeostasis, through hormone-mediated responses [17–20] or even taste perception [21]; the adipocytokine signalling pathway [17, 19]; the vitamin D metabolic pathway [13]; and nervous system development and regulation [22].

The accumulation of evidence for the association of genomic regions with backfat thickness across diverse genetic backgrounds could disentangle which of the reported QTL represent the most prevalent genes and pathways that underlie backfat deposition. In turn, it could also be hypothesized that less prevalent and population-specific associations may derive from variants with larger effects that have been (nearly) fixed in intensely selected populations. In this study, we performed a large genome-wide association study (GWAS) for backfat thickness in eight pig breeding lines of diverse genetic backgrounds, with ~ 15,000 to ~ 55,000 pigs each, for a total of 275,590 pigs. Our main objectives were to determine the genetic architecture of backfat thickness and to identify the main genes and pathways that underlie its genetic variance.

## Methods

### Data

Data comprised 278,112 purebred pigs from eight lines (A to H) of diverse genetic backgrounds (Table 1) from the Pig Improvement Company (PIC; Hendersonville, TN). Breeds of origin of the eight lines included Large White, Landrace, Pietrain, Hampshire, Duroc and synthetic lines. Most pigs were born during the 2008–2017 decade. Backfat thickness was measured by ultrasound in the live pigs at about 145 days of age at the tenth rib. Phenotype values were preadjusted for non-genetic effects (contemporary group, litter, and weight) by line. In total, 2522 outlier preadjusted phenotype values, defined as those outside ± 3 standard deviations of the mean within line, were excluded, and 275,590 records remained for further analyses. Pigs were genotyped with either the GGP-Porcine LD BeadChip with 15 k SNPs or the GGP-Porcine HD BeadChip with 50–80 k SNPs (GeneSeek, Lincoln, NE). We used SNPs that mapped to autosomes based on the reference genome version Sscrofa11.1 and excluded SNPs with a call rate lower than 0.95 and a minor allele frequency lower than 0.01. We also excluded individuals with more than 10% missing genotypes. The remaining SNP genotypes were imputed using multi-locus iterative peeling with the AlphaPeel software [23]. Table 1 summarises the number of individuals and SNPs per line that remained after filtering.

**Table 1:** Number of individuals and SNPs for the eight evaluated lines

Line	Number of individuals	Number of SNPs
A	55,069	69,286
B	53,387	68,499
C	48,752	68,072
D	30,718	60,903
E	28,982	61,135
F	28,499	61,856
G	15,597	64,754
H	14,586	66,437
Total	275,590	71,324

### Genome-wide association study

For each line, we estimated SNP associations by fitting a univariate linear mixed model that accounted for the genomic relationship matrix as:

$$\mathbf{y}= {\mathbf{x}}_{i}{\upbeta }_{i}+\mathbf{u}+\mathbf{e},$$

where $\mathbf{y}$ is the vector of preadjusted phenotypes, ${\mathbf{x}}_{i}$ is the vector of genotypes for the $i$th SNP coded as 0 and 2 if homozygous for either allele or 1 if heterozygous, ${\upbeta }_{i}$ is the additive effect of the $i$th SNP on the trait, $\mathbf{u}\sim N(0,{\upsigma }_{\mathrm{u}}^{2}\mathbf{K})$ is the vector of polygenic effects with the covariance matrix equal to the product of the polygenic additive variance ${\upsigma }_{\mathrm{u}}^{2}$ and the genomic relationship matrix $\mathbf{K}$, and $\mathbf{e}$ is a vector of uncorrelated residuals. The genomic relationship matrix $\mathbf{K}$ was calculated using centred non-standardized SNP genotypes. We used the GEMMA 0.96 software [24] to fit the model. To assess that the GWAS did not have an increased rate of false positives, we inspected the distribution of the p-values in quantile–quantile (Q-Q) plots using the *qqman* R package [25]. We applied a Bonferroni correction for multiple tests and considered SNP associations with a p-value less than 10^–6^, as significant.

For each line, we defined genomic regions of interest that harboured significant SNPs by applying 0.5-Mb flanking regions downstream and upstream of the significant SNP. The genomic regions that overlapped because they arose from nearby significant SNPs within the same line were merged into a single genomic region and those that overlapped across lines were also merged into a single larger genomic region to facilitate comparison across lines.

### SNP-based heritability and genetic variance partitioning by genomic region

To estimate the SNP-based heritability and the genetic variance explained by each genomic region, we fitted a ridge regression model, as implemented in AlphaBayes [26], which uses a Bayesian approach with a Gaussian prior for the SNP effects, a flat prior for the intercept, and a scaled-inverse chi-squared prior for the residual variance. Posterior samples of the SNP effects within each genomic region were obtained from 60,000 Markov-chain Monte Carlo iterations after a burn-in period of 20,000 iterations. In each iteration, the total additive genetic variance was calculated as the variance of breeding values across all individuals. The breeding value of the $j$th individual was calculated as ${\mathrm{BV}}_{j}={\mathbf{x}}_{j}{\mathbf{\upbeta}}$, where ${\mathbf{x}}_{j}$ is the vector of genotypes of all SNPs of the $j$th individual, and ${\mathbf{\upbeta}}$ is the vector of corresponding SNP effects. For each genomic region, regional breeding values were calculated for all individuals using only the subset of SNPs in each genomic region. The variance of the breeding values obtained for each genomic region was calculated and divided by the total additive genetic variance to estimate the proportion of the additive genetic variance explained by the genomic region. The SNP-based heritability was calculated as the total additive genetic variance divided by the phenotypic variance. All breeding values, variances, and variance ratios were calculated in each iteration to obtain posterior distributions for the proportion of the SNP-based heritability and the genetic variance explained by each genomic region. We summarised these posterior distributions by reporting the median value.

### Functional candidate genes and previously reported QTL

The genes located within each genomic region were extracted using the BioMart tool of the Ensembl Genome Browser (Ensembl Genes 100). In order to detect potential functional candidate genes, gene annotation was retrieved from databases of the Gene Ontology project and the KEGG Pathway Database integrated in the Enrichr gene analysis [27].

Data belonging to previously reported QTL that mapped to the reference genome version Sscrofa11.1 were downloaded from the Animal QTLdb [28] (February 2021). QTL entries for traits related to backfat thickness (e.g., average backfat thickness, backfat thickness at last rib, or backfat thickness at last lumbar vertebrae), fat metabolism and deposition (e.g., obesity index, intramuscular fat or triglycerides level), and feed efficiency (e.g., daily feed intake or feed conversion ratio) were selected. Only entries for QTL that were shorter than 5 Mb and that overlapped with the genomic regions found in our GWAS were retained. Enrichment of the genomic regions for QTL terms was tested using the hypergeometric test approach implemented in the GALLO package [29], where the number of QTL entries in the genomic regions identified by the GWAS was compared with the total number of QTL entries for the same term along the whole genome.

## Results

**Fig. 1:**
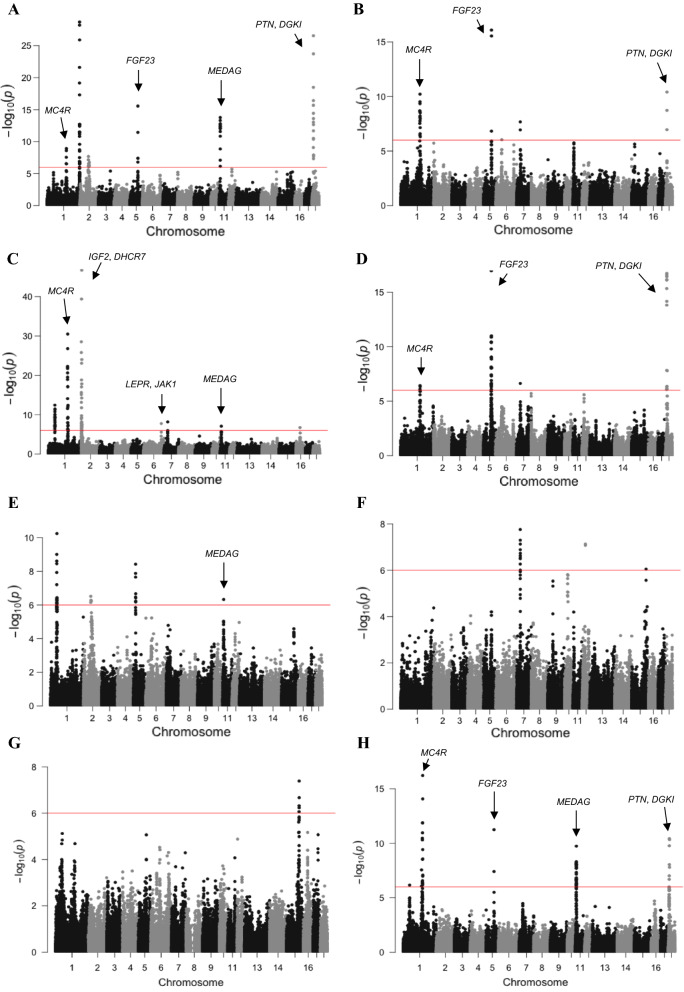
Manhattan plots for the genome-wide association study of backfat thickness for the eight lines. The red line represents the p-value threshold of 10^–6^ used to consider a SNP significant. Reported gene symbols represent the most relevant candidate genes

**Fig. 2:**
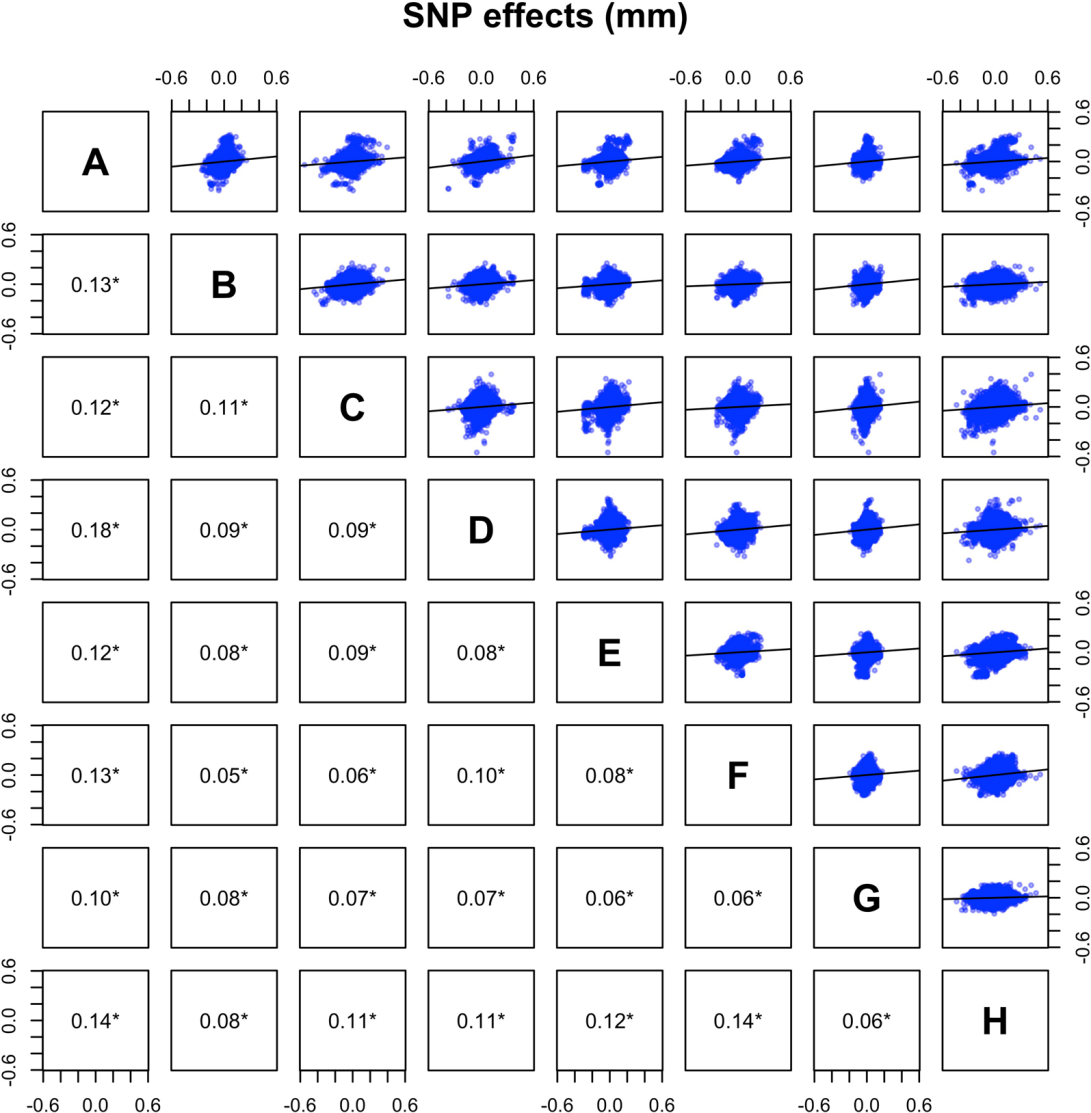
Distribution of estimates of the effects of all SNPs on backfat thickness between each pair of lines. Correlations under the diagonal (asterisk indicates significant correlations, p < 0.05)

**Fig. 3:**
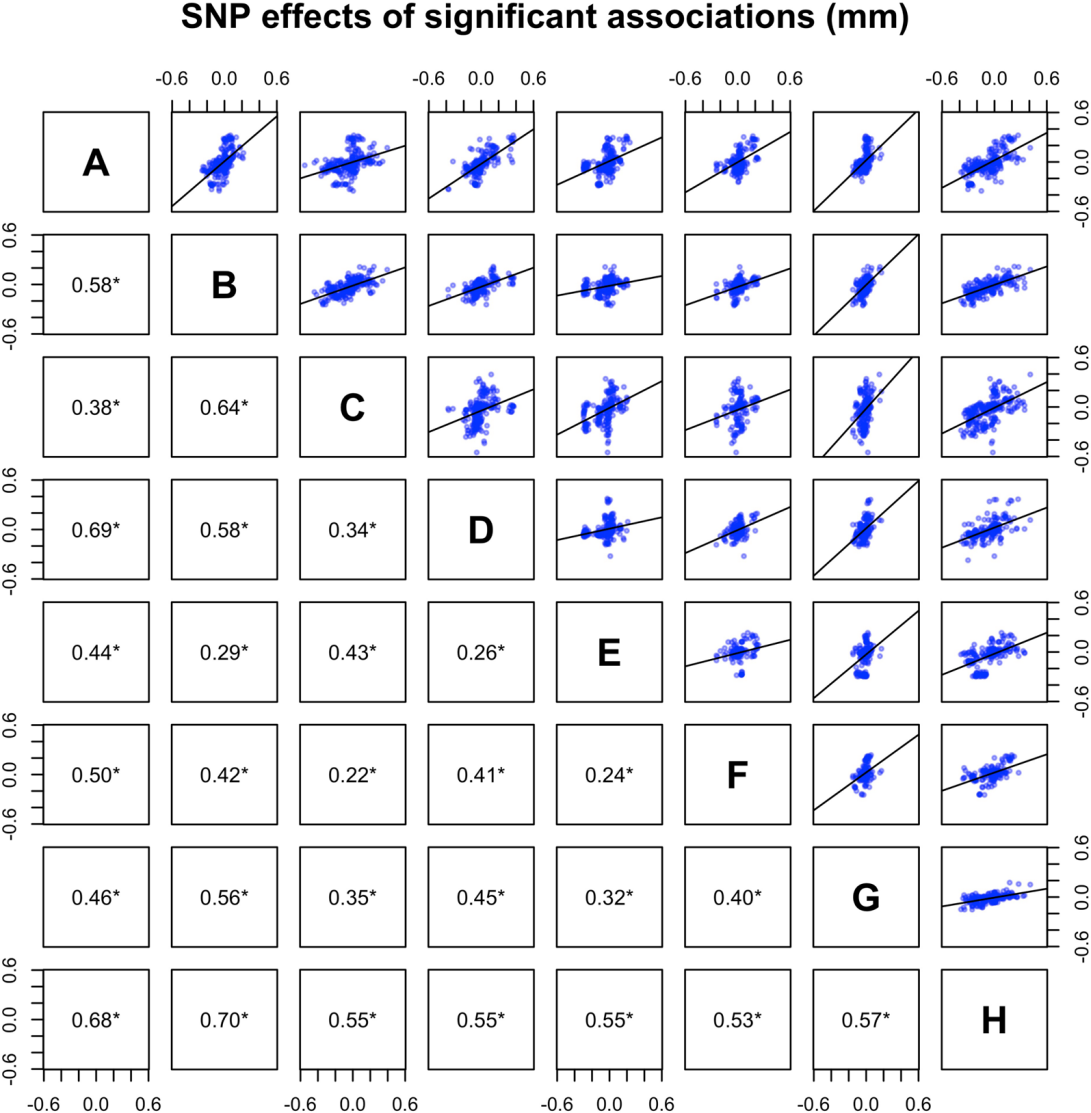
Distribution of estimates of the effects of the 264 significant SNPs on backfat thickness between each pair of lines. Correlations under the diagonal (asterisk indicates significant correlations, p < 0.05)

We found significant genome-wide associations with backfat thickness for 264 SNPs in 27 genomic regions, of which six were detected in three or more lines. Genome-wide associations by line are shown in Fig. 1. The significant SNPs (p < 10^–6^) and their location in the genome are in Table 2. In general, estimates of SNP effects were low to moderate, but a small fraction of SNPs had larger effects of up to 0.55 mm (0.30 additive genetic standard deviation units) [see Additional file 1: Figure S1]. The estimates of SNP effects were largely consistent across lines. The correlations of estimates of SNP effects between lines were positive (0.05 to 0.18 when all SNPs were considered, Fig. 2), and especially high for SNPs that were significant in at least one line (0.22 to 0.70, Fig. 3). The Q-Q plots for each line are in Figure S2 [see Additional file 1: Figure S2].

**Table 2:** Summary of genomic regions significantly associated with backfat thickness and the most significant SNPs for each region

SSC	Position (Mb)	Line	Number of significant SNPs	Most significant SNP			
				Position (bp)	Estimate (SE), mm	P-value	Minor allele frequency
1	*51.17–53.51*^a^	E	19	52,652,849	0.28 (0.04)	5.69 × 10^–11^	0.07
1	*52.74*	H	1	52,740,803	0.25 (0.05)	6.86 × 10^–7^	0.41
1	*52.98–54.13*	C	20	53,262,786	0.27 (0.04)	3.87 × 10^–13^	0.38
1	152.10–152.20	H	2	152,100,725	0.29 (0.05)	2.78 × 10^–8^	0.46
1	*158.31–162.35*	H	18	160,773,437	0.41 (0.05)	6.06 × 10^–17^	0.45
1	*158.36–161.82*	B	15	159,869,511	0.26 (0.04)	6.20 × 10^–11^	0.29
1	*159.54–162.19*	C	21	160,773,437	0.39 (0.03)	3.22 × 10^–31^	0.28
1	*159.70–161.33*	A	5	160,773,437	0.17 (0.03)	1.18 × 10^–9^	0.38
1	*161.07–162.35*	D	3	161,610,871	0.17 (0.03)	3.76 × 10^–7^	0.29
1	163.31–164.83	C	3	163,311,604	0.24 (0.03)	4.24 × 10^–13^	0.34
1	269.18–271.24	A	26	270,408,730	0.25 (0.02)	1.96 × 10^–29^	0.25
2	0.03–4.32	C	32	3,689,100	0.55 (0.04)	1.93 × 10^–47^	0.07
2	60.64–62.25	E	3	60,697,443	0.16 (0.03)	3.07 × 10^–7^	0.27
2	66.01	E	1	66,008,692	0.23 (0.05)	5.82 × 10^–7^	0.09
2	69.12–69.26	A	3	69,257,674	0.31 (0.06)	2.08 × 10^–8^	0.03
2	71.33–71.61	A	2	71,325,641	0.28 (0.06)	2.68 × 10^–7^	0.03
2	73.84–73.92	A	2	73,837,976	0.27 (0.05)	4.69 × 10^–7^	0.03
2	75.75–75.84	A	2	75,750,519	0.27 (0.05)	4.15 × 10^–7^	0.03
2	76.91	A	1	76,905,754	0.27 (0.05)	1.75 × 10^–7^	0.31
5	18.68–19.82	E	9	18,826,228	0.14 (0.02)	3.75 × 10^–9^	0.27
5	*65.30–67.16*	D	20	66,103,958	0.21 (0.02)	1.06 × 10^–17^	0.25
5	*65.89–66.22*	B	3	66,103,958	0.18 (0.02)	7.97 × 10^–17^	0.45
5	*66.00–66.10*	H	2	66,103,958	0.27 (0.04)	5.70 × 10^–12^	0.22
5	*66.10–66.95*	A	5	66,103,958	0.13 (0.02)	2.73 × 10^–16^	0.42
5	69.40	D	1	69,400,164	0.15 (0.03)	8.70 × 10^–9^	0.41
6	47.61	B	1	47,605,459	0.17 (0.04)	9.00 × 10^–7^	0.47
6	147.49	C	1	147,491,028	0.18 (0.03)	1.80 × 10^–8^	0.26
7	*30.10–30.89*	F	10	30,144,081	0.25 (0.04)	1.73 × 10^–8^	0.14
7	*30.32*	C	1	30,317,219	0.22 (0.04)	6.67 × 10^–9^	0.25
7	*30.32–30.33*	B	2	30,317,219	0.17 (0.03)	2.04 × 10^–8^	0.29
7	31.99	D	1	31,986,215	0.20 (0.04)	2.37 × 10^–7^	0.09
11	*7.03–9.57*	H	33	7,946,341	0.33 (0.05)	1.83 × 10^–10^	0.46
11	*7.84*	C	1	7,841,215	0.22 (0.04)	8.68 × 10^–8^	0.17
11	*7.84–8.35*	A	16	7,867,966	0.29 (0.04)	1.64 × 10^–14^	0.06
11	*8.04*	E	1	8,041,891	0.16 (0.03)	4.72 × 10^–7^	0.32
12	25.35–25.37	F	2	25,371,905	0.13 (0.03)	7.33 × 10^–8^	0.18
15	104.07–104.90	G	5	104,902,093	0.14 (0.02)	4.07 × 10^–8^	0.36
15	119.13	F	1	119,128,056	0.15 (0.03)	8.92 × 10^–7^	0.14
16	33.49	C	1	33,493,718	0.14 (0.03)	1.92 × 10^–7^	0.41
18	*8.32–10.66*	H	7	9,460,208	0.35 (0.05)	3.73 × 10^–11^	0.18
18	*9.51–11.78*	D	14	10,578,193	0.37 (0.04)	1.87 × 10^–17^	0.03
18	*10.11–11.78*	A	16	10,578,193	0.33 (0.03)	2.77 × 10^–27^	0.03
18	*10.28–10.58*	B	3	10,578,193	0.15 (0.02)	3.97 × 10^–11^	0.33
18	13.10	D	1	13,102,224	0.32 (0.06)	1.60 × 10^–8^	0.02

^a^Italic type indicates overlapping genomic regions across lines

A region on *Sus scrofa* chromosome (SSC) 1 at ~ 160 Mb (158.31–162.35 Mb) was detected in five lines (lines A, B, C, D and H). Three other regions were detected in four lines: on SSC5 at ~ 66 Mb (65.30–67.16 Mb; lines A, B, D and H), on SSC11 at ~ 8 Mb (7.03–9.57 Mb; lines A, C, E, and H), and on SSC18 at ~ 10 Mb (8.32–11.78 Mb; lines A, B, D and H). Two regions were detected in three lines: on SSC1 at ~ 52 Mb (51.17–54.13 Mb; lines C, E and H) and on SSC7 at ~ 30 Mb (30.10–30.89 Mb; lines B, C and F).

Table 3 shows estimates of SNP-based heritability and of genetic variance by line and the proportion of genetic variance accounted for by each genomic region that harboured significant SNPs. We estimated an average SNP-based heritability of 0.48 across the lines, with estimates by line ranging from 0.30 to 0.58. The significant genomic regions jointly explained from 3.2 to 19.5% of the additive genetic variance of backfat thickness in individual lines. The individual significant genomic regions explained up to 8.0% of the additive genetic variance of backfat thickness. The significant genomic regions also explained up to 1.6% of the additive genetic variance in the lines for which they did not have a statistically significant association with backfat thickness.

**Table 3:** Estimates of SNP-based genetic parameters and percentage of additive genetic variance of backfat thickness explained by the significant genomic regions in each line

SNP-based genetic parameters		A	B	C	D	E	F	G	H
Heritability		0.52	0.51	0.58	0.30	0.53	0.50	0.36	0.54
Additive genetic variance, mm^2^		1.65	2.20	3.41	0.94	1.35	1.47	0.43	2.50
Additive genetic variance by genomic region, %									
SSC	Position, Mb								
1	50.67–54.63	1.10	0.52	*3.17*^a^	0.22	*0.97*	0.10	0.77	*0.98*
1	151.60–157.80	0.11	1.23	0.12	0.08	0.14	0.09	0.15	*0.41*
1	157.81–162.85	*1.85*	*3.34*	*5.33*	*0.54*	0.07	0.05	0.18	*1.24*
1	162.86–165.33	0.09	0.12	*0.24*	0.08	0.06	0.05	0.06	0.06
1	268.68–271.74	*8.02*	0.15	0.27	0.33	0.49	0.26	0.21	0.27
2	0.00–4.82	0.27	0.39	*6.71*	1.07	0.35	0.31	0.21	0.22
2	60.14–67.56	0.04	0.12	0.04	0.13	*0.48*	0.11	0.11	0.13
2	67.57–77.41	*0.29*	0.17	0.12	0.13	0.62	0.10	0.20	0.20
5	18.18–20.32	0.12	0.13	0.17	0.14	*0.95*	0.13	0.10	0.16
5	64.80–67.66	*0.93*	*2.10*	0.10	*1.56*	0.25	0.27	0.32	*0.30*
5	67.67–69.90	0.07	0.21	0.06	*0.34*	0.06	0.05	0.07	0.11
6	47.11–48.11	0.05	*0.13*	0.03	0.05	0.08	0.06	0.03	0.05
6	146.99–147.99	0.07	0.45	*0.49*	0.07	0.10	0.02	0.07	0.08
7	29.60–31.39	0.31	*0.36*	*1.08*	0.04	0.46	*0.57*	0.13	0.93
7	31.40–32.49	0.04	0.21	0.09	*0.02*	0.12	0.04	0.07	0.05
11	6.53–10.07	*0.37*	1.60	*0.84*	0.23	*2.04*	0.32	0.13	*4.25*
12	24.85–25.87	0.19	0.05	0.06	0.07	0.05	*0.05*	0.07	0.05
15	103.57–105.40	0.40	0.04	0.03	0.30	0.05	0.01	*0.37*	0.01
15	118.63–119.63	0.07	0.25	0.13	0.07	0.05	*0.26*	0.02	0.05
16	32.99–33.99	0.09	0.06	*0.14*	0.07	0.05	0.07	0.06	0.06
18	7.82–12.28	*0.28*	*0.86*	0.24	*0.28*	0.19	0.29	0.08	*1.23*
18	12.29–13.60	0.11	0.07	0.05	*0.12*	0.05	0.03	0.00	0.08
Remainder		85.13	87.46	80.48	94.05	92.33	96.77	96.58	89.08

^a^Italic type indicates a significant association of the genomic region found in the GWAS with data from that line

Within the 27 genomic regions, we identified 1219 positional candidate genes, of which 64 are annotated to functions that can be related to fat metabolism (Table 4). The functional annotation of these 64 candidate genes supports a role for energy homeostasis genes in regulating backfat thickness development in pigs, such as *MC4R* on SSC1 at ~ 160.8 Mb (significant association in five lines). Other candidate genes with fat-related functions were identified, such as *MEDAG* on SSC11 at ~ 7.5 Mb, which is involved in adipocyte differentiation and showed a significant association in four lines. While this region did not show significant associations in the other lines, it explained 1.6% of the additive genetic variance in one other of the lines for which the genomic region was not statistically significant. Another example is the genomic region that contains the *IGF2* gene on SSC2 at ~ 1.5 Mb, which is involved in regulation of fat deposition. This region was significantly associated with backfat thickness in only one line but explained 1.1% of the additive genetic variance in at least one other line. The results also suggest that genes involved in phosphate, calcium, and vitamin D homeostasis pathways, such as *FGF23* on SSC5 at ~ 66.0 Mb, contribute to backfat thickness development.

**Table﻿ ﻿4:** Functional candidate genes and previously reported QTL for backfat-related traits in the significant genomic regions

SSC	Position, Mb	Lines	Number of QTL	QTL traits	Number of positional candidate genes	Genes with related annotated functions^b^
1	50.67–54.63	C, E, H	11	BFT*, DFI* (7), IMF, OI, RFI	43	*CYB5R4*^4,5^
1	151.60–152.70	H	2	BFT*, DFI*	5	–
1	157.81–162.85	A, B, C, D, H	45	BFT* (3), BF10R (4), BFLR* (5), BFR, DFI* (13), FCR (2), IMF, LMP* (10), OI (6)	53	*KDSR*^2^, *MC4R*^4^
1	162.86–165.33	C	1	LMP*	45	*ATP8B1*^2,8^, *SLC51B*^2,8^, *CILP*^5^, *HACD3*^2,3^
1	268.68–271.74	A	6	AFW, BF10R (2), BFMB, BFW, FCR	83	*SLC27A4*^2,3,5^, *DOLK*^2^, *DOLPP1*^2^, *CRAT*^3^
2	0.00–4.82	C	64	BFT* (19), AFW, BF10R (3), BF34R (3), BFFR, BFLL (4), BFLR* (7), BFR (7), BFW, FCR (6), LMP* (12)	138	*PTDSS2*^2^, *HRAS*^5,6^, *PNPLA2*^2,3^, *BRSK2*^5^, *IGF2*^4^, *INS*^1,2,4,5^, *DHCR7*^7^, *FGF19*^4^, *CPT1A*^2,3,5,6^, *GAL*^5^, *LRP5*^1,5^, *CHKA*^2^
2	60.14–62.75	E	–	–	87	*SLC27A1*^3,5^, *SIN3B*^2^
2	65.51–66.51	E	–	–	42	*GCDH*^3^
2	68.62–69.76	A	–	–	53	*RDH8*^7^, *CARM1*^1,2^
2	70.83–72.11	A	1	IMF	52	*ANGPTL4*^2,3^, *CERS4*^2^, *INSR*^2,4,5^
2	73.34–74.42	A	–	–	25	*PLIN5*^1,2,3^
2	75.25–76.34	A	3	BFT* (2), LMP*	35	*S1PR4*^2^, *GNA15*^2^, *GNA11*^5^
2	76.41–77.41	A	–	–	47	*MKNK2*^5^, *ATP8B3*^2^, *STK11*^4,6^, *ABCA7*^2^
5	18.18–20.32	E	–	–	83	*SOAT2*^2,7^, *CALCOCO1*^7^
5	64.80–67.66	A, B, D, H	6	BFT*, BF34R (2), IMF(2), TGL	49	*FGF23*^7^
5	68.90–69.90	D	2	BFT*, BFLR*	16	*HDHD5*^2^
6	47.11–48.11	B	2	BFMD, IMF	59	*SIRT2*^1^, *ZFP36*^1^
6	146.99–147.99	C	14	BFT* (2), BF10R (5), BF34R, BFLL, BFLR* (3), IMF(2)	11	*JAK1*^6^, *LEPR*^4,6^ (at 146.80–146.90 Mb)
7	29.60–31.39	B, C, F	23	BFT* (7), ADAR, AFW, BF34R, BF67R, BFFR, BFLR* (3), BFMB, BFR (2), FCR, LMP*, SSFT (3)	46	*DAXX*^7^, *ITPR3*^5^, *PPARD*^1,2,3^
7	31.49–32.39	D	1	FCR	23	*PNPLA1*^2^, *CDKN1A*^6^
11	6.53–10.07	A, C, E, H	2	BFT*, RFI	62	*ALOX5AP*^3^, *MEDAG*^1^
12	24.85–25.87	F	2	IMF (2)	35	*GIP*^5^, *PHB*^7^, *NGFR*^4^
15	103.57–105.40	G	–	–	32	–
15	118.63–119.63	F	3	BFT*, DFI*, LMP*	9	*IGFBP2*^5^, *IGFBP5*^5^
16	32.99–33.99	C	10	FCR (10)	6	–
18	7.82–12.28	A, B, D, H	8	BFT* (2), BFLR*, IMF (5)	71	*AGK*^2^, *ATP6V0A4*^5^, *AKR1D1*^7,8^, *DGKI*^2^, *PTN*^1,7^
18	12.60–13.60	D	1	BFR	9	–

*BFT* average backfat thickness, *ADAR* adipocyte area, *AFW* abdominal fat weight, *BF10R* backfat thickness at tenth rib, *BF34R* backfat thickness between the third and fourth rib, *BF67R* backfat thickness between the sixth and seventh rib, *BFFR* backfat thickness at first rib, *BFLL* backfat thickness at last lumbar, *BFLR* backfat thickness at last rib, *BFMB* backfat thickness at mid-back, *BFMD* backfat thickness above muscle dorsi, *BFR* backfat thickness at rump, *BFW* backfat weight, *DFI* daily feed intake, *FCR* feed conversion ratio; *IMF* intramuscular fat content, *LMP* lean meat percentage, *OI* obesity index, *RFI* residual feed intake, *SSFT* shoulder subcutaneous fat thickness, *TGL* triglyceride level. Within parentheses: number of entries if there were more than one. *Enriched QTL traits (p < 10^–4^)

Superscript numbers: ^1^Adipogenesis pathways; ^2^Lipid metabolism pathways; ^3^Fatty acid metabolism pathways; ^4^Energy homeostasis pathways; ^5^Insulin signalling pathways; ^6^Adipocytokines signalling pathways; ^7^Steroid hormone and vitamin D metabolism pathways; ^8^Bile acid metabolism

Table 4 also shows 207 previously published QTL entries for 21 fatness and feed efficiency traits that overlapped 20 of the regions with significant SNPs. The detected genomic regions were enriched for previous QTL entries for average backfat thickness, backfat thickness at last rib, daily feed intake, and lean meat percentage (p < 10^–4^). For seven of the regions we found no previously reported QTL, and for 12 of the regions we found previously reported QTL for fat metabolism and deposition or for feed efficiency traits but not for backfat thickness traits. These 12 regions showed a significant association in only one of the lines and, in general, explained a low proportion of the genetic variance.

## Discussion

To our knowledge, this is the largest-to-date GWAS for backfat thickness in pigs. We report results from eight large populations, which ranged from ~ 15,000 to ~ 55,000 genotyped pigs and differed in breed of origin and selection history. Large sample sizes are required for high power of GWAS and, thus, this dataset provides valuable insight into the genetic architecture of backfat thickness and the main genes and pathways that underlie its genetic variance.

We found significant associations for 27 genomic regions, of which one region was detected in five out of the eight lines, three regions in four lines and two regions in three lines. Moreover, some of these genomic regions explained a relatively large proportion of the additive genetic variance of backfat thickness in lines for which the GWAS detected no significant association. The genomic regions that were detected in five or four of the lines contained candidate genes *MC4R*, *MEDAG*, *FGF23*, *DGKI*, and *PTN*. Together with the candidate genes that were found in the other genomic regions, the results support the involvement of energy homeostasis, adipogenesis, fatty acid metabolism, and insulin signalling pathways, and suggest the contribution of other metabolic pathways, which are less well understood, to genetic variation for backfat thickness in pigs, such as the phosphate, calcium, and vitamin D homeostasis pathways.

In the light of these findings, we will focus our discussion on: (1) the genetic architecture of backfat thickness, (2) the role of energy homeostasis genes on backfat thickness, (3) the role of adipogenesis, fatty acid metabolism, and insulin signalling genes on backfat thickness, and (4) the role of phosphate, calcium, and vitamin D homeostasis genes on backfat thickness.

### Genetic architecture of backfat thickness

The results of the detected SNP associations and genetic variance partitioning confirm the polygenic architecture of backfat thickness, with many loci with small individual effects and only a small fraction of SNPs with larger effects. The effect of the significant SNPs was largely maintained across the eight studied lines. Our moderate-to-high estimates for SNP-based heritability are in the range of previous SNP-based estimates in purebred Duroc lines (0.31 [30] or 0.37 [19]), Landrace (0.47) [31], Large White (0.35) [31], and Pietrain (0.39) [31], and even in Pietrain crosses with Large White x Landrace (0.45), Meishan (0.73) and wild boar (0.42) [32].

We detected several genomic regions that significantly affected backfat thickness and individually explained up to 8.0% of the genetic variance for the trait. Genomic regions that were detected in more than one line or that explained a large proportion of genetic variance generally overlapped with entries for backfat-related QTL with evidence of enrichment and with candidate genes with plausible annotated functions. The fact that a genomic region was detected in a single line or in multiple lines was not always related to the proportion of genetic variance explained by the region in those lines. Moreover, some genomic regions explained a relevant proportion of genetic variance in lines for which that genomic region was not significant. Previous reports in a Duroc population estimated that a single genomic region, in particular the region on SSC6 where the *LEPR* gene is located, could explain up to 19.8% of the additive genetic variance of backfat thickness [17]. We observed no instances of any single genomic region that explained such a high percentage of the genetic variance. In that same population, the genomic region on SSC1, which includes the *MC4R* gene, explained 1.1% of the additive genetic variance [17]. These two regions explained up to 0.5% (*LEPR* region) and 5.3% (*MC4R* region) of the additive genetic variance in the lines studied here.

After accounting for significant genomic regions from the GWAS, the majority of the additive genetic variance remained distributed across non-significantly associated genomic regions; in the genetic variance partitioning analysis, the residual polygenic term due to these non-significant SNPs explained from 80.5 to 96.8% of the additive genetic variance in each line. Thus, most of the genetic variance is explained by minor loci that were not detected in the GWAS. Taken together, these results confirm that backfat thickness has a polygenic architecture, although some major genes that agree with previous studies [13, 17, 22] contribute large proportions of the genetic variance of the trait in some lines. In that regard, shifting towards an omnigenic model [33] may provide a more suitable conceptualisation of the genetic architecture of backfat thickness.

Previous studies have estimated that dominance effects account for 4 to 15% of the phenotypic variance, and imprinting effects for 1 to 3% [30, 31]. Indeed, major genes such as *LEPR* and *IGF2* have been reported to have dominance [19, 34] and imprinting [8] effects, respectively, on backfat thickness. While non-additive effects may be of interest for understanding genetic or physiological mechanisms, they have limited applications in breeding practices [35]. Statistical additive variance captures a fraction of the non-additive effects. Thus, our study focused on the additive variance, because it is the fraction of genetic variance that is most commonly targeted in GWAS and most useful for directional selection.

### Energy homeostasis genes

The GWAS results support the role of energy homeostasis genes for genetic variation in backfat thickness in pigs. Significant SNPs in the genomic region on SSC1 at ~ 160 Mb were found in five lines. The functional candidate gene *MC4R* is located in this region. Energy homeostasis in mammals is a feedback system that balances energy intake and expenditure. The melanocortin-4 receptor that is encoded by *MC4R* has been described as a critical coordinator of mammalian energy homeostasis and body weight [36]. Mutations in *MC4R* are well known in pigs and are involved in regulating appetite [11, 37]. The role of mutations in *MC4R* in human monogenic obesity was described in the late 1990s [38, 39] and has also been described to affect variation in fatness, growth, and feed intake in different pig breeds [10, 20, 37, 40–42].

Another key regulator of feed intake and energy homeostasis is the *LEPR* gene. A mutation in this gene was described in an Iberian $\times$ Landrace cross [43] that reduced leptin signalling and resulted in greater feed intake and therefore greater carcass fat content [44]. Similar findings were found in Duroc pigs [19] and in a Duroc $\times$ Landrace/Large White cross [37]. In fact, studies in pigs confirmed that serum leptin concentration is an effective predictor of fat accumulation [4]. We only found one significant SNP near this gene in one of the eight lines, on SSC6 at 147.5 Mb, which is only 0.6 Mb from the *LEPR* gene at 146.8–146.9 Mb. Non-significance of this region in the other lines could be the result of intense selection for feed efficiency in the studied lines [34]. A study on signatures of selection in a Duroc line that was selected for increased intramuscular fat content, with a correlated response for backfat thickness, revealed greater extended haplotype homozygosity in this region compared to a control line [45]. The candidate genes *JAK1* and *LEPROT* also map to this region. In fact, the significant SNP at 147.5 Mb is located in an intron of the *JAK1* gene. The annotated functions of *JAK1* and its effect on backfat thickness are difficult to disentangle from the effects of other genes, such as *LEPR* or *LEPROT*.

### Adipogenesis, fatty acid metabolism, and insulin signalling genes

The results of the GWAS also support that other pathways are involved in the development of backfat in pigs, such as adipogenesis, fatty acid metabolism, and insulin signalling. Adipogenesis involves differentiation from pre-adipocytes to adipocytes. The region on SSC11 at ~ 8 Mb, which was identified in four lines, contains the adipogenic gene *MEDAG*, which promotes adipocyte differentiation and lipid accumulation in mature adipocytes [46] and was shown to be upregulated in fat compared to lean pigs [14]. The same region also contains the gene *ALOX5AP*, which is involved in subcutaneous fat deposition in pigs [14].

The gene *PTN*, which is in the region on SSC18 at ~ 10 Mb that was identified in four lines, has a role in a signalling pathway that negatively regulates adipogenesis [47]. Recent in vitro studies in mice demonstrated that *PTN* plays an essential role in the dynamics of adipose lipid turnover and plasticity, as it preserves insulin sensitivity and regulates energy metabolism and thermogenesis [48]. The gene *DGKI*, which maps to this same region, has been reported to be under positive selection in polar bears and could be related to the development of corporal fat to provide thermal isolation [49].

Other genes with annotated functions related to adipogenesis that were implicated in this GWAS include genes that have been previously associated with body fat content in other species, such as *LRP5* (body fat distribution in humans) [50], *BRSK2* (abdominal fat in chicken) [51], and *DOLK* (subcutaneous fat in lambs) [52]. Of these, *LRP5* and *BRSK2* are in the 0.00–4.82 Mb genomic region on SSC2. Although we detected a significant association for this region in only one line, several GWAS on backfat thickness in pigs have revealed a significant association of this genomic region with backfat thickness, average daily gain, and meat-to-fat ratio in diverse genetic backgrounds, from F_2_ populations derived from breeds such as Pietrain, Large White and Landrace [32] to crosses of Iberian pigs with Landrace, Pietrain, and Duroc [13], and many QTL reports support these findings. This region is gene-rich and includes many candidate genes, such as the *INS* gene, which encodes insulin that regulates blood glucose levels, promotes cell fat storage, and regulates the activity of enzymes that intervene in lipid metabolism [53], and the *IGF2* gene, which encodes the insulin-like growth factor 2, and is widely considered as a major candidate gene for muscle mass and fat deposition in pigs [7–9, 22, 32, 54, 55]. However, it has been suggested that other genes in the same region could have an effect on backfat thickness independent of *IGF2*, such as *CTSD*, which encodes a protease [55], and genes related to fatty acid metabolism, such as *CPT1A*, which is involved in the oxidation of long-chain fatty acids, which are the main storage lipids that form backfat. The *FADS1*, *FADS2*, and *FADS3* genes, which encode fatty acid desaturases, are located near this region (at ~ 9.6–9.7 Mb). Genes related to fatty acid oxidation, such as *PLAAT3* (at ~ 8.4 Mb), and fibroblast growth factor genes, such as *FGF19* [13, 32]*,* have also been considered as potential candidate genes for growth and fat deposition traits in this region.

The *PLIN5* gene, which is located in the significant SSC2 region at ~ 74.3 Mb, is involved in the control of intracellular lipid deposition and some results indicate that it may be involved in regulation of the expression of hormone-sensitive lipase [56]. Other genes of the perilipin family have also been associated to differences in backfat thickness [57].

Across the genomic regions we identified candidate genes with functions in bile acid metabolism. Bile, which is predominantly formed by steroid bile acids synthesized from cholesterol, breaks down fat into monoglyceride and fatty acids that can be absorbed by the digestive tract. The *SOAT2* gene [58] is involved in biliary cholesterol metabolism and the *SLC51B* [59], *ATP8B1* [60], and *AKR1D1* [61] genes are involved in bile formation. This is not the first study that pointed to bile acid metabolism genes as candidates for backfat thickness in pigs through the mechanism of lipid absorption in the intestine [15], although the *BAAT* gene that was proposed in that previous study was not in any of the significant genomic regions identified in our study.

### Phosphate, calcium and vitamin D homeostasis genes

Due to the biological complexity of polygenic traits, some metabolic pathways that have been less explored could nonetheless also have an impact on backfat thickness. For instance, calcium [62, 63] and phosphate [64, 65] have been linked to adipocyte differentiation and lipid metabolism in human and rats. We found significant SNPs in the genomic region on SSC5 at ~ 66 Mb in four lines. The candidate gene *FGF23* is located in this region. This gene is responsible for phosphate homeostasis through a pathway that involves feedback regulation by phosphate, calcium, and vitamin D [66–68]. To our knowledge, this is the first GWAS that detects a significant association of the *FGF23* genomic region with backfat thickness in pigs, although this gene was previously linked to mineral utilization and homeostasis in Landrace pigs (not significant after correction for multiple testing) in relation with bone development [69]. However, there is some evidence for a potential mechanistic link between *FGF23* and adiposity. Lean adipose tissue secretes adiponectin, which causes a significant reduction in the expression of *FGF23* in osteocytes [70], while expanded adipose tissue secretes leptin, which increases *FGF23* expression in osteocytes [71]. In humans, clinical studies support a potential role of *FGF23* signalling in the metabolic status of individuals, including insulin resistance, dyslipidemia, and obesity [72, 73]. For instance, it has been reported that the level of FGF23 in blood was higher in obese compared to normal-weight adolescents [74] and that this level was positively correlated with fat mass and triglyceride levels [75].

The genomic region on SSC18 at ~ 10 Mb that includes the *PTN* gene was detected in the same four lines as the SSC5 genomic region that contains *FGF23*. As well as having a role in regulation of adipogenesis (as discussed above) [47], the *PTN* gene is also involved in vitamin D-dependent regulation of calcium and phosphate homeostasis [76]. To our knowledge, there is, however, no evidence for any interaction between *PTN* and *FGF23* [77].

The *DHCR7* gene, which is located in the genomic region on SSC2 at ~ 2.4 Mb close to the *IGF2* and other genes, has also been associated with backfat thickness in different pig populations [13, 32]. The enzyme encoded by *DHCR7* catalyses the conversion of 7-dehydrocholesterol to cholesterol, the final step in the production of cholesterol. 7-dehydrocholesterol is also a precursor for vitamin D and, therefore, *DHCR7* plays an important role in vitamin D metabolism in humans [78, 79]. However, the role of vitamin D in adiposity is unclear. Meta-analyses in humans have shown that, although low vitamin D levels are commonly observed in obese people, which is likely due to sequestration of the fat-soluble vitamin in adipose tissue, vitamin D supplementation did not consistently reduce body weight in clinical trials [80, 81]. Although this gene is in a genomic region that contains other candidate genes with more plausible annotated functions, the characterization and validation of *DHCR7* and other genes such as *FGF23* and *PTN* with functions related to phosphate, calcium and vitamin D homeostasis could shed new light on genetic variation for backfat thickness.

## Conclusions

Our GWAS results obtained on 275,590 pigs from lines with diverse genetic backgrounds confirmed the polygenic architecture of backfat thickness and the importance of genes associated with energy homeostasis, adipogenesis, fatty acid metabolism, and insulin signalling pathways for fat deposition in pigs. The results also suggested that genes involved in phosphate, calcium, and vitamin D homeostasis contribute to backfat development. While the association with backfat thickness of genes such as *MC4R*, *IGF2*, and *LEPR* has been studied during the last decades, the genomic regions detected here also contained more novel candidate genes, such as *DHCR7*, *FGF23*, *MEDAG*, *DGKI*, and *PTN*. We quantified that these and other genomic regions could individually contribute up to 8.0% of the genetic variance in the studied lines. The characterization of genes with annotated functions that are not well understood is challenging but can shed new light on the genetic and physiological mechanisms that control adiposity. Further research on these candidate genes is encouraged in order to identify putative causal genomic variants that contribute to the genetic variance in backfat thickness in pigs and to assess their potential application in swine breeding programmes.

## Supplementary Information

**Additional file 1: Figure S1.** Distribution of the SNP effects on backfat thickness (mm) in the eight pig lines. **Figure S2.** Q-Q plots for the genome-wide association study of backfat thickness for the eight pig lines.

## Data Availability

The software packages AlphaPeel and AlphaBayes are available from https://github.com/AlphaGenes. The datasets generated and analysed in this study are derived from the Genus PIC breeding programme and not publicly available.
