# Supplementary material for: Genetic architecture and major genes for backfat thickness in pig lines of diverse genetic backgrounds
Source: Genet Sel Evol. 2021 Sep 22;53:76. doi: 10.1186/s12711-021-00671-w (PMC8459476; doi:10.1186/s12711-021-00671-w)
Supplement: Supplementary file 1 — Additional file 1: Figure S1. Distribution of the SNP effects on backfat thickness (mm) in the eight pig lines. Figure S2. Q-Q plots for the genome-wide association study of backfat thickness for the eight pig lines. [file 12711_2021_671_MOESM1_ESM.pdf]

Additional file 1

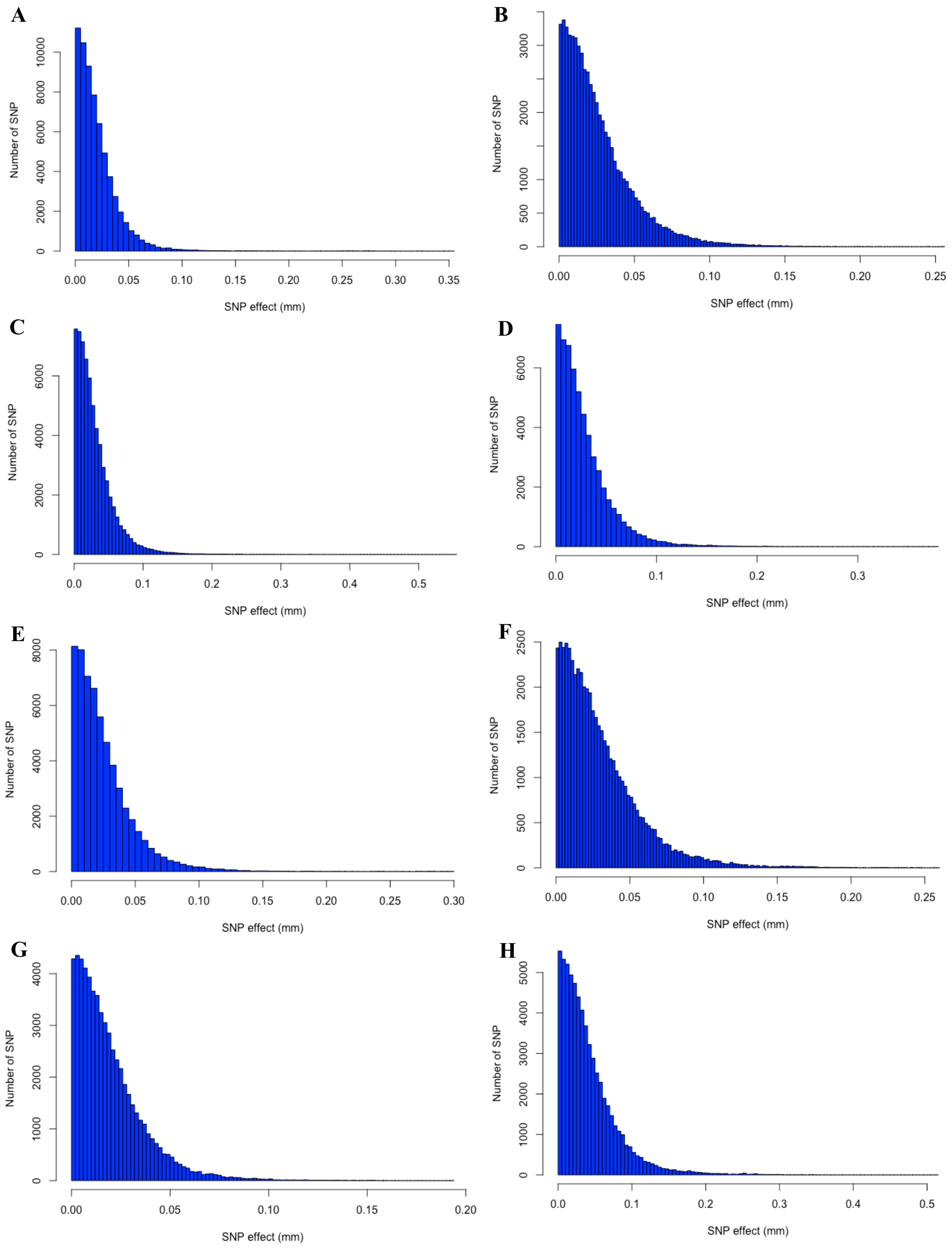

Figure S1. Distribution of the SNP effects on backfat thickness (mm) in the eight pig lines.

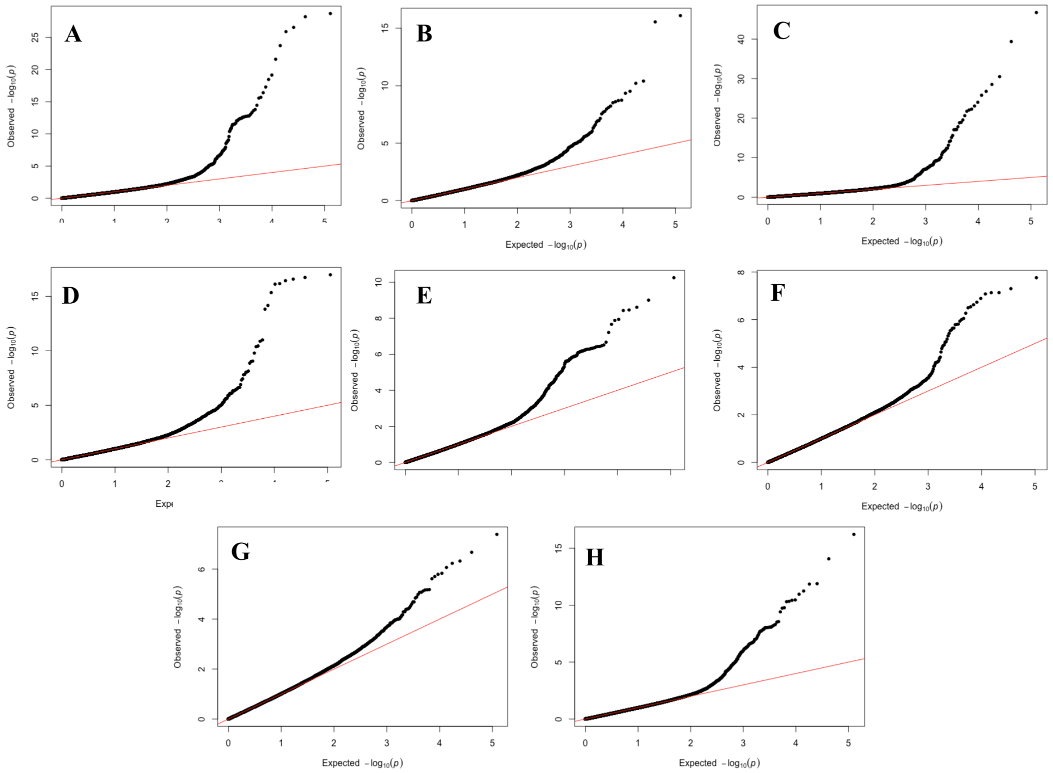

Figure S2. Q-Q plots for the genome-wide association study of backfat thickness for the eight pig lines.
